# Idiopathic Ventricular Fibrillation: Diagnosis, Ablation of Triggers, Gaps in Knowledge, and Future Directions

**DOI:** 10.19102/icrm.2020.110604

**Published:** 2020-06-15

**Authors:** Soufian T. Almahameed, Elizabeth S. Kaufman

**Affiliations:** ^1^Heart and Vascular Center, MetroHealth Campus of Case Western Reserve University, Cleveland, OH, USA

**Keywords:** Ablation, premature ventricular complexes, Purkinje, ventricular fibrillation

## Abstract

Idiopathic ventricular fibrillation (IVF) is a diagnosis of exclusion made when no underlying cause is identified in a cardiac arrest survivor. Although the frequency of this diagnosis has declined over time due to advances in diagnostic techniques, it remains a substantial cause of sudden cardiac arrest. Further, IVF tends to recur. This article reviews the criteria for diagnosis, patient characteristics, the two primary arrhythmic phenotypes—short-coupled variant of torsades de pointes and recurrent paroxysmal IVF—and the electrophysiologic features, treatment, and ablation of premature ventricular complexes that can trigger IVF.

## Introduction

The annual incidence of deaths due to sudden cardiac arrest (SCA) in the United States has been estimated as approximately 300,000 to 450,000.^1,2^ Although a recent increase in the proportion of deaths from pulseless electrical activity and bradycardia has occurred, ventricular arrhythmias remain responsible for most cases of SCA.^3–5^ Data from the Cardiac Arrest Survivors with Preserved Ejection Fraction Registry reveal an underlying cardiovascular structural or molecular cause for cardiac arrest in more than half of patients without overt structural heart disease, leaving 44% of these patients as unexplained cardiac arrest survivors.^6^

Although ischemic cardiomyopathy still accounts for the majority of SCA,^7^ advances in the treatment of coronary disease have reduced the proportion of SCA occurring as a result of ischemia. At the same time, advances in imaging have enabled the detection of subtle instances of structural heart disease, including myocardial fibrosis related to sarcoidosis, myocarditis, and other arrhythmogenic cardiomyopathies. Even so, an important minority of patients with ventricular fibrillation (VF) have no detectable structural heart disease nor an established molecular mechanism and are considered to have idiopathic ventricular fibrillation (IVF).

IVF accounts for 5% to 10% of survivors of out-of-hospital cardiac arrest.^8–11^ Because recurrent VF events are frequent and poorly predictable, placement of an implantable cardioverter-defibrillator (ICD) is considered first-line therapy for patients with IVF.^12^ The recurrent arrhythmic event rate and appropriate ICD shock rate is very high among IVF patients, estimated to be 31% in the meta-analysis by Ozaydin et al., with a 3.1% mortality rate during an average of five years of follow-up.^13^ Although ICDs reduce mortality in survivors of IVF, they do not address the IVF substrate; hence, in the absence of substrate modification, recurrent VF, syncope, cardiac arrest, and ICD shocks occur.

In this review, we will discuss the proposed criteria for IVF diagnosis, previous reports of premature ventricular complex (PVC)-triggered IVF (PVC-VF) or short-coupled torsades de pointes (sc-Tdp), diagnostic strategies to identify potential candidate PVC-VF triggers for radiofrequency ablation, and future directions on screening for IVF substrate in SCA survivors. We will also define gaps in knowledge, including the identification of individuals at risk for IVF.

## Case presentation

**Figure 1:**
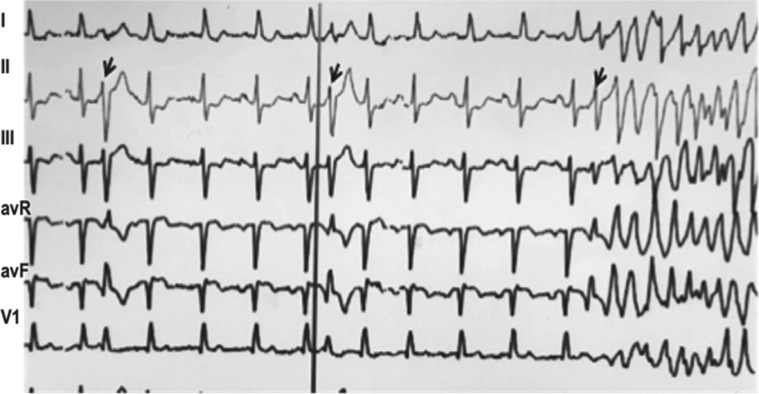
Example of a presenting arrhythmia on telemetry tracing showing sinus rhythm with unifocal short-coupled PVC initiating PMVT.

A 56-year-old white female with a history of resuscitated SCA and an ICD placed for secondary prevention presented having experienced multiple ICD shocks due to polymorphic ventricular tachycardia (PMVT). A 12-lead electrocardiogram (ECG) showed normal sinus rhythm and frequent unifocal narrow PVCs (QRS duration: 130 ms) with no ST-segment elevation or depression. The QT interval was normal and there were no early or late repolarization abnormalities observed. Her telemetry showed frequent unifocal narrow PVCs with a right-bundle left superior axis (RBLS) morphology triggering PMVT **(Figure 1)**. Echocardiography showed preserved cardiac function. The patient underwent coronary angiography, which revealed a 90% right coronary artery lesion; it was treated successfully with primary coronary intervention with a drug-eluting stent. She continued to have the same unifocal PVCs triggering runs of PMVT, which subsided within 48 hours after treatment with mexiletine 150 mg three times daily.

**Figure 2:**
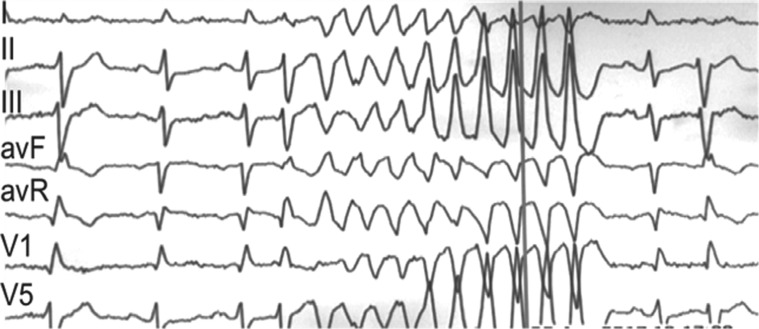
Telemetry tracing of short-coupled unifocal PVCs initiating PMVT at the second presentation.

She presented again several months later with multiple syncopal episodes and ICD shocks. Device interrogation revealed PMVT, while telemetry showed recurrent PMVT salvos with uniform initiating PVCs with the same morphology (RBLS) as seen previously **(Figure 2)**. Coronary angiography showed a widely patent RCA stent.

**Figure 3:**
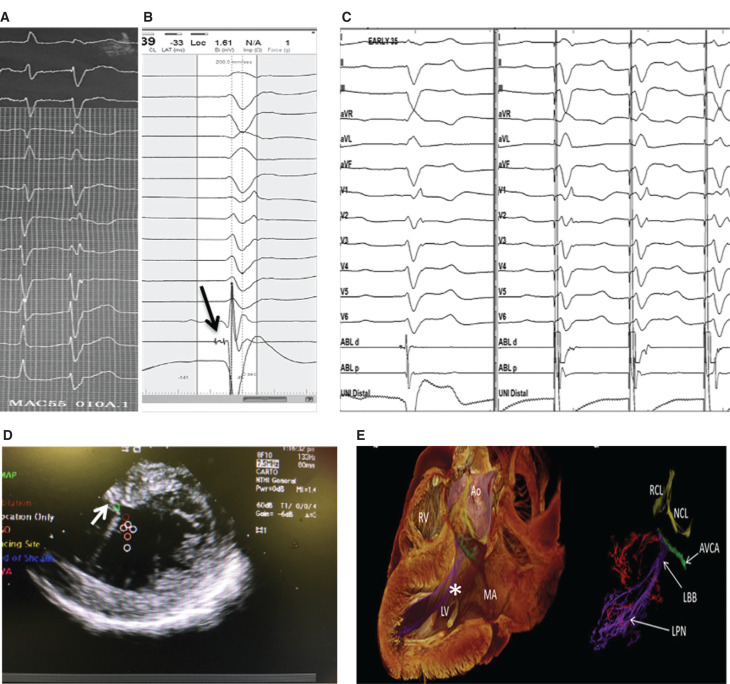
**A:** Resting 12-lead ECG of sinus beat followed by PVC. Note there is no evidence of ischemia or early repolarization at the 12-lead ECG sinus beat. **B:** Intracardiac mapping showing a 45-ms presystolic fractionated abnormal distal left posterior fascicle Purkinje potential (arrow) at the site of origin of the culprit PVC (sweep speed: 200 ms). **C:** Clinical PVC on a recording system and pacemapping at the distal Purkinje site of origin. **D:** Real-time intracardiac echo image during ablation at the PVC site of origin (arrow) at the posterior fascicle of the LB. The tip of the ablation catheter is depicted in green. **E:** High-resolution CT scan illustrates the three-dimensional anatomy of the human conduction system; note, in particular, the location of the left anterior and left posterior fascicles in relation to the site (star) of successful ablation. LV: left ventricle; MA: mitral annulus; RV: right ventricle; AO: aorta; RCL: right coronary leaflet; NCL: noncoronary leaflet; AVCA: atrioventricular conduction axis; LPN: left Purkinje network. Figure 3E reprinted with permission from Stephenson RS, Atkinson A, Kottas P, et al. High resolution 3-dimensional imaging of the human cardiac conduction system from microanatomy to mathematical modeling. *Sci Rep*. 2017;7(1):7188.

The patient underwent electrophysiology study and successful mapping and ablation of a Purkinje PVC trigger arising from the distal left posterior fascicle **(Figure 3)**. Mexiletine was discontinued and no recurrent PMVT or ICD shocks have been seen during follow-up.

### Criteria for idiopathic ventricular fibrillation diagnosis and initial workup in survivors

**Figure 4:**
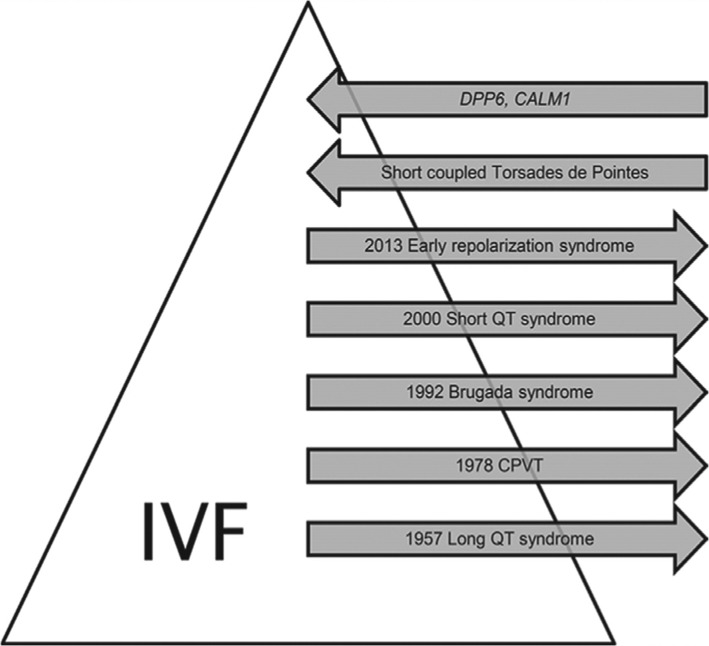
Schematic illustration of the evolution of the diagnosis of IVF. CALM1: calmodulin 1 gene mutation; CPVT: catecholaminergic polymorphic ventricular tachycardia; DPP6: Dutch DDP6 risk haplotype associated with sudden cardiac death. Reprinted with permission from Visser M, van der Heijden JF, Doevendans PA, Loh P, Wilde AA, Hassink RJ. Idiopathic ventricular fibrillation: the struggle for definition, diagnosis, and follow-up. *Circ Arrhythm Electrophysiol*. 2016;9(5):e003817.

As defined by the Heart Rhythm Society/European Heart Rhythm Association/Asia-Pacific Heart Rhythm Society expert consensus statement on inherited primary arrhythmia syndromes, IVF is “a resuscitated cardiac arrest … with documentation of VF, in whom known cardiac, respiratory, metabolic, and toxicologic etiologies have been excluded through clinical evaluation.”^14^ The consensus statement acknowledges that the frequency of diagnosis of IVF has declined over the last four decades due to improvements in diagnostic techniques that can unmask an underlying structural or molecular etiology in cases previously considered to be IVF. **Figure 4** shows the decline in IVF diagnosis over time, primarily owing to the significant discoveries and modern technologies that have identified underlying molecular mechanisms of VF, rendering it secondary to a known arrhythmia syndrome.^15^

**Figure 5:**
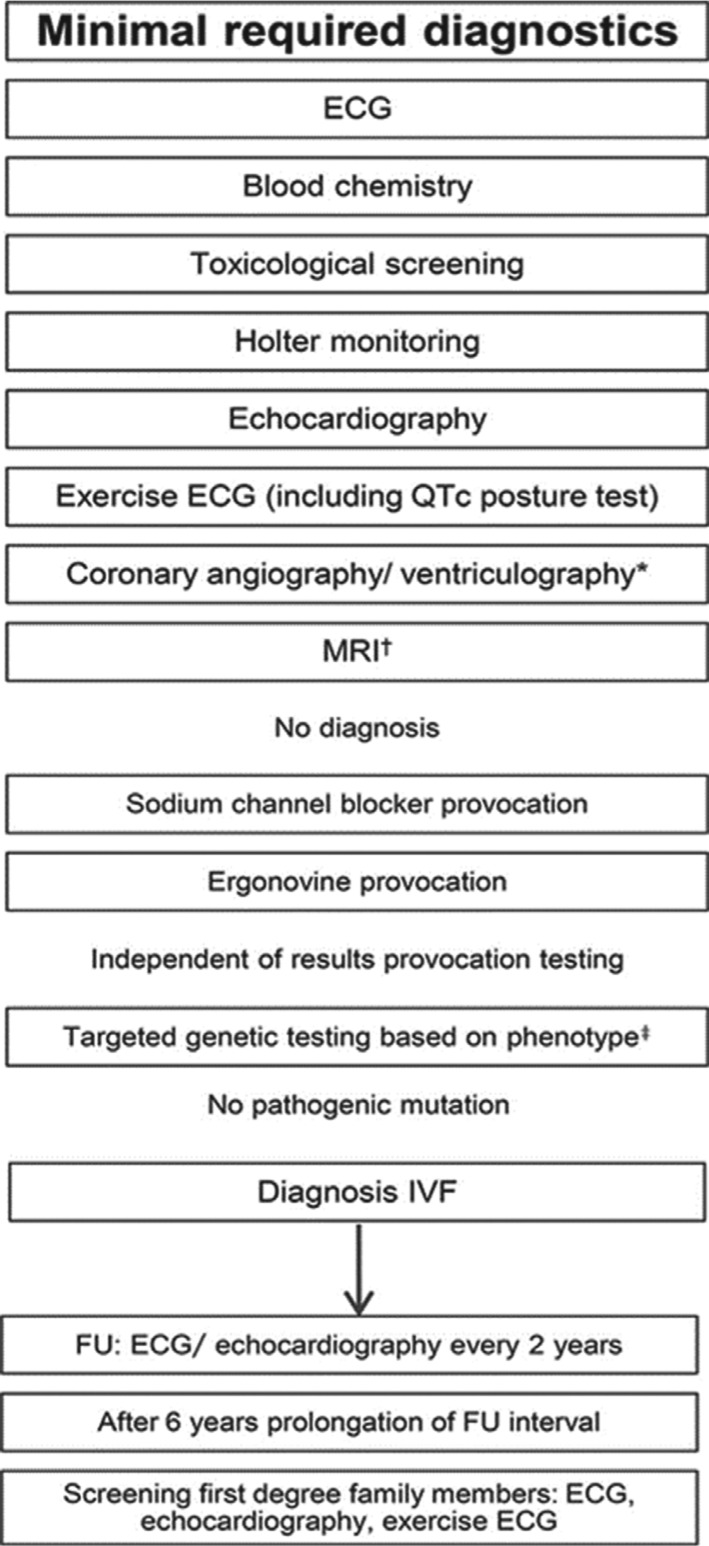
Proposed flowchart for the diagnosis and follow-up of patients with IVF. *In young patients (< 45 years) without risk factors for coronary artery disease, coronary CT, or magnetic resonance angiography are alternative diagnostic tools by which to exclude coronary artery disease. ^†^A proposed acquisition protocol for cardiac MRI is available in the Data Supplement of Visser et al.^15^^‡^Proposed genetic testing consisting of a basic panel of *SCN5A*, the most common long-QT genes (*KCNQ1* and *KCNH2*), *RyR2*, and *CALM1* in patients with exercise-induced VF. In patients with a negative phenotype, *SCN5A*, *KCNQ1*, and *KCNH2* are recommended*.* FU: follow-up. Reprinted with permission from Visser M, van der Heijden JF, Doevendans PA, Loh P, Wilde AA, Hassink RJ. Idiopathic ventricular fibrillation: the struggle for definition, diagnosis, and follow-up. *Circ Arrhythm Electrophysiol*. 2016;9(5):e003817.

Because IVF is the diagnosis of exclusion in patients presenting with SCA, a comprehensive and stepwise initial clinical workup is needed to exclude any cardiac (structural or molecular), respiratory, metabolic, and toxicologic underlying causes **(Figure 5)**.^15^ A meticulous inspection of the 12-lead ECG should be done to exclude any pathologic ECG findings including the early-repolarization J-wave syndrome phenotypes or QT syndromes. Significant clues suggesting the underlying mechanism can be found upon analysis of telemetry and ambulatory monitor tracings. The use of provocation tests to survey for concealed sodium or potassium channel mutation should be considered when no apparent structural [normal two-dimensional echo, coronary angiogram, and cardiac magnetic resonance imaging (MRI)] or molecular mechanism is identified. In the search for underlying causes of VF, the importance of exercise testing cannot be overemphasized, since a substantial number of patients with VF have undiagnosed catecholaminergic PMVT.^16^ Exercise testing may also unmask occult long-QT syndrome. A corrected QT interval of more than 445 ms at four minutes of recovery from exercise has been used to detect patients with long-QT syndrome types 1 and 2.^17^ The infusion of epinephrine can also be helpful for the diagnosis of catecholaminergic PMVT [characterized by increasing ventricular ectopy, bidirectional ventricular tachycardia (VT), or polymorphic VT] or occult long-QT syndrome, particularly type 1. Details of epinephrine infusion and interpretation can be found in a paper by Obeyesekere et al.^18^

With suspicion of Brugada syndrome, a sodium-channel blocker can be administered to provoke a type I Brugada syndrome ECG pattern. In the United States, this is often performed with an infusion of 1 g or 10 to 15 mg/kg of procainamide over 30 minutes while obtaining serial ECG recordings with the inclusion of “high” anterior precordial leads placed two intercostal spaces above the usual position.^18^

The exclusion of a coronary etiology, whether acquired disease or congenital anomaly, can be accomplished with coronary angiography or, particularly in young patients, with coronary computed tomography (CT) or cardiac MRI.

**Figure 6:**
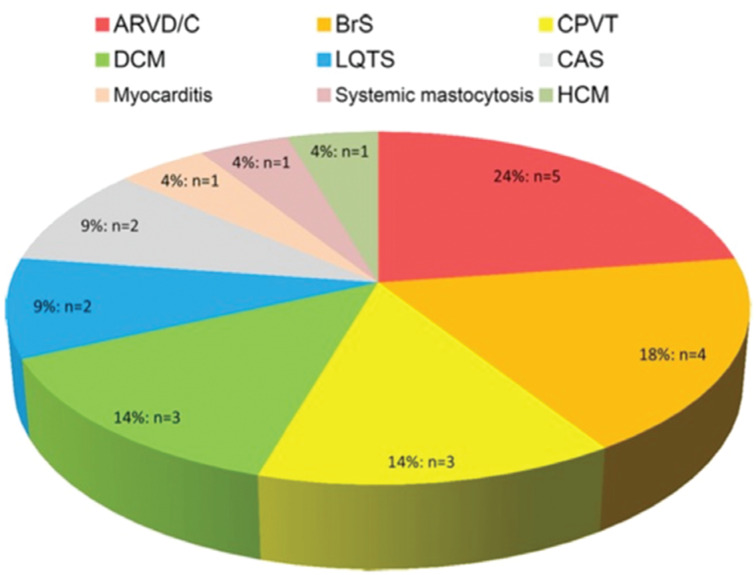
Overview of specific diagnoses that were revealed during follow-up. ARVD/C: arrhythmogenic right ventricular dysplasia/cardiomyopathy; Brs: Brugada syndrome; CAS: coronary artery spasm; CPVT: catecholaminergic polymorphic ventricular tachycardia; DCM: dilated cardiomyopathy; HCM: hypertrophic cardiomyopathy; LQTS: long-QT syndrome. Reprinted with permission from Visser M, van der Heijden JF, van der Smagt JJ, et al. Long-term outcome of patients initially diagnosed with idiopathic ventricular fibrillation: a descriptive study. *Circ Arrhythm Electrophysiol*. 2016;9(10):e004258.

The recommendation for follow-up testing **(Figure 5)** is crucial for several reasons. First, patients may develop a clinical phenotype over time. Second, advances in diagnosis (imaging and molecular) and in treatments continue to occur. Third, a follow-up visit offers the opportunity to ensure that family members are appropriately screened, particularly when an underlying diagnosis is identified. In a long-term (median: 10.2 years) follow-up study of patients believed to have IVF, a diagnosis potentially explaining the VF was eventually found in 21% of patients. Notably, about half of these cases were detected by genetic testing. The distribution of diagnoses is shown in **Figure 6**.^19^

### Characteristics of idiopathic ventricular fibrillation patients

In a large meta-analysis of 23 studies including 639 subjects with IVF, the mean age ranged from 33 years to 51 years, and 70% (n = 449) of patients were male.^13^ Over approximately five years, the risk of ventricular arrhythmia recurrence was 31% and that of mortality was 3.1%. Electrophysiologic testing was not predictive of recurrence. In another long-term IVF-cohort follow-up study, the median age at the time of the index event was 40.4 years and 54% of participants were male.^19^ Symptoms prior to cardiac arrest consisted of palpitations in 14% of the patients and syncope in 20% of the patients. A family history of sudden cardiac death (SCD) was noted in 5% of the patients. VF occurred at rest in 61%, with mild activity in 18%, and with exercise in 21% of patients. During an average follow-up of 5.3 years, 29% of the patients received appropriate ICD therapy. The results of electrophysiologic testing were not predictive of recurrent events.

### Patterns of abnormal ventricular rhythm in idiopathic ventricular fibrillation

Two characteristic patterns of IVF can be recognized: sc-Tdp and recurrent paroxysmal primary IVF (PIVF). In both of these patterns, arrhythmia may be initiated by “culprit” or “triggering” PVCs (ie, PVC-VF). Multiform or unifocal PVC-VF can be seen in subjects with IVF, either acutely or chronically. PVC triggers are defined when these PVCs repeatedly initiate VF or VT and have an identical morphology with markedly short coupling interval (CI) (usually < 400 ms) to the last sinus beat. PVC triggers are seen typically at the time of presentation of the index arrhythmia, syncope, resuscitated cardiac arrest, or ICD shock. The exact prevalence of PVC triggers among subjects with IVF is not known. PVC-VF behaviors and frequency rates in these patients are often unpredictable: when present, culprit PVCs tend to especially appear in the immediate aftermath of VF, regardless of whether it is a first IVF or recurrent IVF event.^20^

We henceforth discuss in greater detail the sc-Tdp, PIVF, and PVCs that can trigger IVF. Additionally, we review available case reports and case series of IVF patients undergoing electrophysiology study with and without ablation of PVC-VF. The baseline characteristics of IVF patients are summarized in **Table 1**. The electrophysiology study characteristics of PVC triggers and ablation outcomes are summarized in **Table 2**.

### Short-coupled variant of torsades de pointes

**Figure 7:**
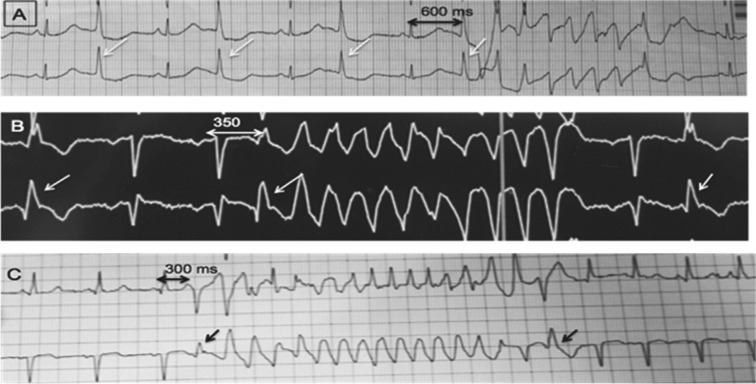
Patterns of PMVT and VF initiation with uniform PVC based on CI between the first initiating beat (uniform PVC trigger) and the preceding normal sinus beat. **A:** Uniform narrow PVCs (arrow) in a patient with long-QT interval initiating PMVT (classical Tdp) with long CI at 600 ms. **B:** Uniform PVCs (arrow) in a patient with no structural heart disease initiating PMVT (sc-Tdp) with short CI at 350 ms. **C:** Uniform PVCs initiating a brief run of PIVF.

sc-Tdp differs from the classic form of Tdp that occurs in patients with congenital or acquired QT-interval prolongation. In patients with long-QT syndrome, the initial beat of Tdp typically has a long CI, usually greater than 500 ms. In contrast, in patients with sc-Tdp, PMVT is initiated with a remarkably short CI **(Figure 7)**.

Initial clinical reports on PMVT and VF in young patients emerged as early as 1966.^21,22^ Leenhardt et al. were the first to describe sc-Tdp in 1994 as a new syndrome associated with sudden death in young and otherwise healthy individuals.^23^ Leenhardt et al. studied 14 patients (mean age: 43.6 ± 10 years) with no structural heart disease who presented between 1972 and 1991, all of whom experienced syncope due to sc-Tdp. Remarkably, only one patient required resuscitation on presentation, indicating that the sc-Tdp arrhythmia was mostly self-terminating. A family history of SCD was present in four (30%) of these patients and the mean CI of the initiating beat was 245 ms ± 28 ms (range: 200–300 ms). In nine (64%) of the 14 patients, this initiating beat exhibited a left bundle (LB) branch (LBB) pattern and left superior axis morphology consistent with an apical RV site of origin. All patients underwent an electrophysiology study, where up to three extra-stimuli were given at different drive cycle lengths of 600 ms, 500 ms, and 400 ms, respectively. The baseline electrophysiologic parameters were normal. The clinical arrhythmia (sc-Tdp) could not be reproduced with programmed stimulation. Only one long coupling Tdp could be reproducibly induced with doubles. High-dose verapamil (360–720 mg/day) was used to suppress PVC triggers in sc-Tdp and was deemed acutely efficacious when it resulted in prolonging the PVC CI as well as decreasing the frequency of PVCs. Among those who were treated with verapamil, no recurrence was seen in six patients over a mean follow-up period of seven years.

Viskin et al.^24^ reported a case series of three patients (mean age: 55 years) who presented with recurrent syncope/PMVT (one patient), near-syncope/PMVT (one patient), and cardiac arrest (one patient) and were all found to have ECG findings consistent with sc-Tdp as defined by Leenhardt et al. All three patients had palpitations due to frequent PVCs; in the two patients with syncope/PMVT, the symptomatic PVCs predated the PMVT event by 10 to 12 years. All three patients were identified to have PVCs of LBI morphology triggering PMVT with a mean CI of 350 ms ± 20 ms to the initiation of sc-Tdp.

The CI in our case presented above was 350 ms and, in retrospect, the finding of significant single-vessel coronary artery disease was incidental. The ECG findings were also consistent with sc-Tdp, despite the history of resuscitated cardiac arrest at initial presentation.

### Recurrent paroxysmal primary idiopathic ventricular fibrillation

VF is a disorganized arrhythmia with a “spontaneous” onset. It rarely self-terminates and is fatal if allowed to persist. Identifiable PVC-VF triggers are present in a subset of successfully resuscitated patients with IVF, typically when they present with ICD shocks due to recurrent VF—hence the term PIVF. Haïssaguerre et al.^20^ studied 27 patients (mean age: 41 ± 14 years) who presented with resuscitated recurrent episodes of IVF. Twenty-three (85%) of these patients had an ICD implanted previously, while a family history of SCD was present in six (22%) patients. The PVC-VF trigger morphology was a right bundle (RB) branch pattern with a very narrow QRS duration at 115 ms ± 11 ms in approximately 40% of the patients and an LBB pattern with wide QRS duration of approximately 145 ms in roughly 50% of the patients. Both LB and RB PVC triggers could be seen in approximately 10% of patients. An LB inferior axis [right ventricular outflow tract (RVOT) morphology] was seen in four patients. Interestingly, the PVC-VF triggers were seen persistently in eight (30%) patients (including all four RVOT morphology patients) but intermittently in 19 (70%) of the patients. The mean CI for PVC-VF triggers measured 297 ms ± 41 ms. An interpolated PVC trigger occurred in 63% of patients and no long–short initiating sequence was observed in any of the patients.

### Electrocardiographic and clinical features of benign versus malignant premature ventricular complexes

“Idiopathic” PVCs arising from the RVOT (LB inferior axis morphology with precordial transition at or after V3) in patients with no structural heart disease are usually—but not always—benign. Noda et al.^25^ described a series of 101 patients with idiopathic VT originating from the RVOT who underwent electrophysiology study and radiofrequency ablation of RVOT VT. Approximately 16% of these patients had also manifested spontaneous VF or PMVT prior to ablation. Patients with these malignant arrhythmias were more likely to have a history of syncope (69% versus 18%; p = 0.0001). The same group reanalyzed these data in a subset of 16 patients with both monomorphic VT (MMVT) and PMVT or VF and the MMVT was faster in these patients relative to among those with MMVT only (273 ± 23 ms versus 328 ± 65 ms; p = 0.0001).^26^ Viskin et al.^24^ compared the CI of PVC triggers in patients with benign RVOT VT (n = 38), sc-Tdp (n = 3), and IVF (n = 12) and found that the CI of the initiating PVC was significantly longer in the benign RVOT VT population (427 ± 76 ms versus 340 ± 30 ms versus 300 ± 40 ms; p < 0.001).

In contrast with idiopathic RVOT PVCs, to the best of our knowledge, there are no reports of idiopathic (non–Purkinje-related) LVOT PVCs (inferior axis morphology, precordial transition before V3) triggering IVF or Tdp, nor has there been a published comparison of the CI of PVC triggering benign LVOT VT and that triggering malignant LVOT VT or IVF. The vast majority of reported PVC-VF triggers are found to arise from the distal Purkinje and, therefore, have a discernibly different morphology than that of LVOT PVC. In addition to the very short CI of left-sided PVC-VF (280 ± 26 ms), the QRS duration is remarkably short (126 ± 18 ms) and could be of any axis (intermediate, superior, or inferior).^20^

### Electrophysiologic characteristics, treatment, and ablation of premature ventricular complex triggers

Haïssaguerre et al. identified the distal Purkinje system as the site of origin of PVC-VF triggers in the majority of their patients with PIVF; in a few individuals, PVC-VF triggers arose from the RVOT.^20^ When mapping PVC-VF, a distal Purkinje site of origin is defined if the Purkinje–ventricular interval coinciding with sinus beats at that site is short (11 ± 5 ms). Conversely, a proximal Purkinje is defined when the Purkinje–ventricular interval coinciding with sinus beats exceeds 15 ms. The Purkinje potential coinciding with PVC-VF at the site of origin was markedly presystolic [earlier for the left-side Purkinje site of origin (38 ± 28 ms) compared with the right-side Purkinje of origin (19 ± 10 ms)]. Radiofrequency application at the Purkinje site of origin typically resulted in the induction of ventricular arrhythmia and, rarely, VF with subsequent termination. The PVC-VF triggers were eliminated and the Purkinje potential was abolished at the conclusion of ablation. These patients were followed for 24 months ± 28 months and 89% experienced no recurrence of VF, syncope, or SCD. In a larger series of 38 patients^27^ who underwent IVF ablation in a similar fashion, 31 (82%) patients remained free of VF recurrence at five years of follow-up. The other seven patients showed VF recurrence within a median of four months of follow-up. Interestingly, a transient ipsilateral conduction delay at the PVC-VF Purkinje of origin was a very strong predictor of VF-free survival (p < 0.0001). The electrophysiology study characteristics of PVC triggers and ablation outcomes from different reports of sc-Tdp and PIVF are summarized in **Table 2**.

**Figure 8:**
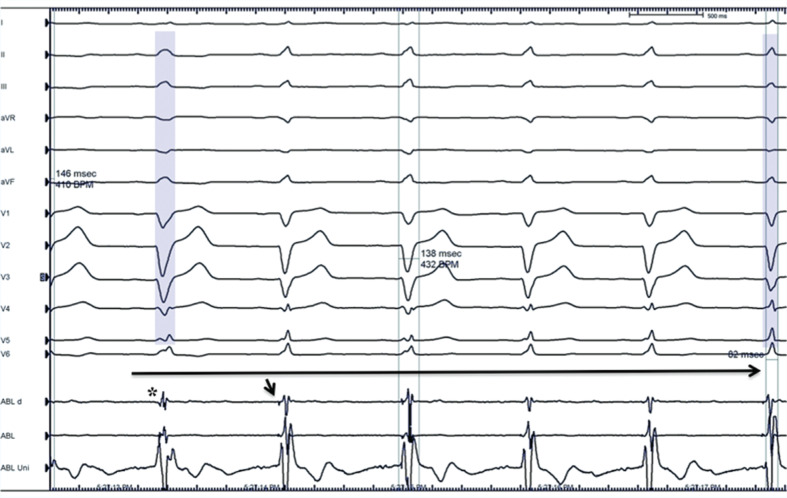
From left to right, the disappearance of distal bundle distal Purkinje potential during catheter manipulation–induced LBB block (star), followed by the resolution of LBB block and the reemergence of the distal Purkinje potential (arrow).

Since the vast majority (93%) of PVC-VF triggers arise from the distal Purkinje system, special attention during mapping is needed to avoid inducing bundle branch block during catheter manipulation, as this could lead to the disappearance of a relevant Purkinje potential at the site of origin of PVC-VF as well as during mapping in sinus rhythm, thus limiting the effectiveness of PVC-VF mapping and ablation **(Figure 8)**.

**Figure 9:**
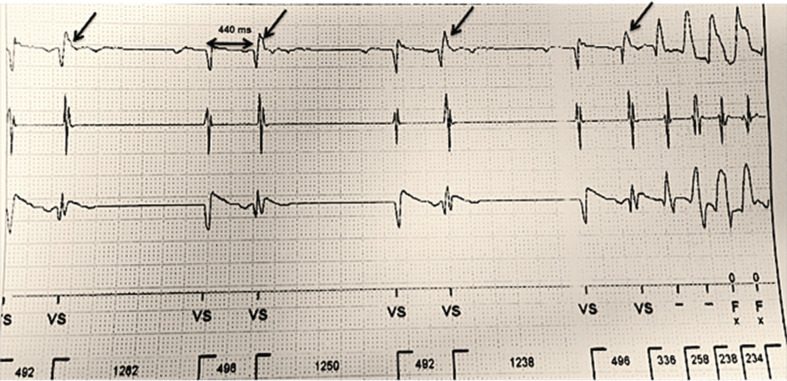
Device interrogation showing uniform PVC-VF triggers.

The prevalence of PVC-IVF triggers in patients with resuscitated VF arrest and ICD shocks due to VF is yet to be determined. In view of the proven short- and long-term efficacy of ablation, however, it is important to search for potential PVC-VF triggers in the immediate aftermath of the event using every available tool, including telemetry monitoring, Holter monitoring, and device interrogation **(Figure 9)**.^28^

### Mechanisms of premature ventricular complex triggers: evidence and speculation

In addition to being the most common site of origin for PVC-VF triggers in patients with IVF, Purkinje PVC-VF triggers have been mapped and successfully ablated in patients with channelopathy including long-QT syndrome,^29,30^ catecholaminergic PMVT,^31^ and early-repolarization syndrome.^32^ Purkinje PVC-VF triggers have also been mapped and ablated in patients with structural heart disease including ischemic heart disease,^33–37^ hypertrophic cardiomyopathy,^38^ left ventricular noncompaction,^39^ amyloidosis,^40^ dilated cardiomyopathy,^41^ myocarditis,^42^ and valvular heart disease.^43^

The Purkinje system is an endocardial structure in humans and constitutes 1% to 2% of the myocardium mass. Given that the distal Purkinje system is overwhelmingly the source of PVC-VF triggers in patients with and without structural or molecular abnormalities, one should suspect distal Purkinje arrhythmogenic disease (DPAD), diffuse or focal and idiopathic or organic, as the common pathway for the initiation of VF.

### Generation of premature ventricular complex triggers

Although Purkinje cells and myocytes originate from the same primary cell lineage, there are many transcriptional, anatomic, and functional differences between the two cellular lines. These differences could, in part, help explain this unique distal Purkinje susceptibility for VF arrhythmogenicity in patients with and without structural heart disease. The distal Purkinje arborization–ventricular muscle junction has dual perfusion through the systemic and cavital blood.^44^ Therefore, Purkinje cells can survive in an anaerobic environment. Purkinje cells express Cx40, a high-conductance channel protein, in addition to Cx43, and therefore exhibit a faster conduction velocity relative to the working myocytes that primarily express Cx43.^45,46^ Purkinje cells contain atrioventricular nodal–like cell properties and, hence, are capable of producing pacemaker-like automaticity.^47,48^ Purkinje cells in canine models express a triple calcium-release channel apparatus in the endoplasmic reticulum (IP_3_R, RyR_2_, and RyR_3_), in contrast with the one-channel apparatus (RyR_2_) present in the ventricular myocytes.^49^ This may account for the high prevalence of triggered activity in Purkinje cells leading to Purkinje ectopics, facilitated by early or delayed afterdepolarizations.^49,50^ In addition to their proven role in triggering VF in humans, evidence from animal studies and computer models suggests that Purkinje cells may be responsible for sustaining VF early after its initiation.^51,52^ Further investigations are needed to better understand the functional, molecular, or anatomic substrate underlying these fatal rhythm phenotypes.

## Summary and future directions

It is likely that future research will unmask the underlying functional, molecular, or structural substrate for IVF in its two remaining phenotypes, sc-Tdp (often presenting with syncope) and PIVF (often presenting with ICD shocks). Until then, it is crucial for clinicians and investigators to be mindful of the now well-recognized role of PVC-VF triggers and to make efforts to further understand their clinical characteristics, prevalence, and diagnosis. The identification and ablation of these PVC-VF triggers, when present, can significantly reduce the likelihood of VF recurrence and its associated morbidities such as syncope, ICD shocks, cardiac arrest, and sudden death. It is of paramount importance that these triggers be captured by surface 12-lead ECGs (preferred), telemetry, or device interrogation upon presentation since they may disappear shortly after the event. For this reason, electrophysiologic study and ablation may be considered during the index presentation in those patients with recurrent drug-refractory sc-Tdp or PIVF. More investigation is needed to better understand DPAD as a clinical and pathologic entity that affects patients with and without manifest structural heart disease. It will also be important to further study PVC characteristics in order to discern and differentiate “malignant” from “benign” PVCs in a patient with a history of syncope. We propose that PVCs in patients with unexplained syncope be categorized as high or low risk according to the PVC morphology (narrow or wide), CI (short or long), and PVC periodicity around the event (persistent or intermittent) **(Table 3)**. We propose that future ICD technology should incorporate a PVC morphologic recognition algorithm to help identify culprit PVC electrograms in patients presenting with ICD shocks for recurrent PMVT or VT. Finally, a noninvasive assessment of DPAD based on the detection of concealed or manifest Purkinje activities may help shed more light on the individual risk and prediction of VF in high-risk patients.

## Figures and Tables

**Table 1:** Baseline Characteristics of IVF Patients in Published Reports

Study	Number of Patients	Age at Presentation	Presentation	PVC Trigger Morphology	PVC Trigger QRS Duration (ms)*	PVC Trigger CI
Leenhardt et al.^23^	14	34.6 ± 10 years	Syncope due to sc-Tdp	LBLS	NA	245 ± 28 ms
Takatsuki et al.^53^	1	49 years	Palpitation, syncope, VF at EP study	LBLI	NA	320 ms
Haïssaguerre et al.^20^	27	41 ± 14 years	VF arrest	LBI (n = 4)	145 ± 12	355 ± 30 ms
				LBS (n = 10)	126 ± 18	280 ± 26 ms
				RB (n = 9)		
				RB and LB (n = 4)		
Leenhardt et al.^23^	14	34.6 ± 10 years	Syncope due to sc-Tdp	LBLS	NA	245 ± 28 ms
Viskin et al.^24^	3	48 ± 11 years	Syncope/PMVT in 2, VF in 1	LBI	NA	340 ± 30 ms
Takatsuki et al.^53^	1	49 years	Palpitation, syncope, VF at EP study	LBLI	NA	320 ms
Saliba et al.^54^	1	41 years	Seizure due to PMVT	LBLS	145	240 ms
Betts et al.^55^	1	27 years	VF arrest	LBI	NA	250 ms
Noda et al.^25^	16	39 ± 10 years	Presyncope and syncope due to VF (n = 5) or PMVT (n = 11)	LBI	NA	409 ± 62 ms
Kohsaka et al.^56^	1	21 years	VF arrest	LBLS	NA	280 ms

CI: coupling interval; EP: electrophysiology; LB: left bundle; LBI: left bundle inferior axis; LBLI: left bundle left inferior axis; LBLS: left bundle left superior axis; LBS: left bundle superior axis; NA: not available; PMVT: polymorphic ventricular tachycardia; PVC: premature ventricular complex; RB: right bundle; sc-Tdp: short-coupled variant of torsades de pointes; VF: ventricular fibrillation.

*Measured by the authors from ECGs in the published manuscript when available.

**Table 2:** Electrophysiology Study Characteristics of PVC Triggers and Ablation Outcomes from Different Reports of sc-Tdp and PIVF

Study	Number of Patients	PVC Trigger Morphology	PVC Trigger SOO	Distal Purkinje PVC Trigger at SOO	Ablation and Follow-up (As Reported)
Haïssaguerre et al.^20^	27	RB (n = 10)	LV Purkinje (n = 10)	46 ± 29 ms	Success rate: 89%; no syncope, sudden death, or recurrence of VF at 24 ± 28 months
		RB and LB (n = 4)	LV/RV Purkinje (n = 4)		
		LB (n = 9)	RV Purkinje (n = 9)	19 ± 10 ms	
		LBI (n = 4)	RVOT myocardium (n = 4)	NA	
Viskin et al.^24^	3	LBI	RVOT myocardium	NA	No recurrence at 8 years, 2 years, and 5 months
Takatsuki et al.^53^	1	LBLI	RVOT myocardium	NA	Successful; no recurrence; follow-up duration not specified
Saliba et al.^54^	1	LBLS	Purkinje RV	60 ms	Successful; no recurrence at 6 months
Betts et al.^55^	1	LBI	RVOT myocardium	NA	Successful; no recurrence at 11 months
Noda et al.^25^	16	LBI	RVOT myocardium	NA	81% successful and 19% partially successful; one patient received ICD; no recurrences of syncope, VF, or SCD at 54 ± 39 months
Kohsaka et al.^56^	1	LBLS	RV Purkinje	72 ms	Successful; no recurrence at 12 months

ICD: implantable cardioverter-defibrillator; LB: left bundle; LBI: left bundle inferior axis; LBLI: left bundle left inferior axis; LBLS: left bundle left superior axis; LV: left ventricle; NA: not available; PIVF: paroxysmal primary idiopathic ventricular fibrillation; PVC: premature ventricular complex; RB: right bundle; RV: right ventricle; RVOT: right ventricular outflow tract; SCD: sudden cardiac death; sc-Tdp: short-coupled variant of torsades de pointes; SOO: ; VF: ventricular fibrillation.

**Table 3:** Features of Low and High Risks for PVCs in Patients with Unexplained Syncope and Apparently Normal Hearts

Low-risk PVCs	
–	Present with longstanding palpitations
–	Persistent
–	Long CI (; 500 ms)
Malignant PVCs	
–	Periodic, with frequency concentrated in the aftermath of VF or PMVT presentation
–	Short CI (< 400 ms)^20,23,25^
–	RB pattern with narrow QRS duration < 140 ms^20^
–	LB pattern superior axis with late precordial transition (RV apex)^20,23^

CI: coupling interval; LB: left bundle; PMVT: polymorphic ventricular tachycardia; PVC: premature ventricular complex; RB: right bundle; RV: right ventricle; VF: ventricular fibrillation.
